# Supplementary material for: Bacteroides vesicles promote functional alterations in the gut microbiota composition
Source: Microbiol Spectr. 2024 Sep 30;12(11):e00636-24. doi: 10.1128/spectrum.00636-24 (PMC11537023; doi:10.1128/spectrum.00636-24)
Supplement: Fig. S3 — Histological examination of intestinal tissue from DSS group. [file spectrum.00636-24-s0003.pdf]

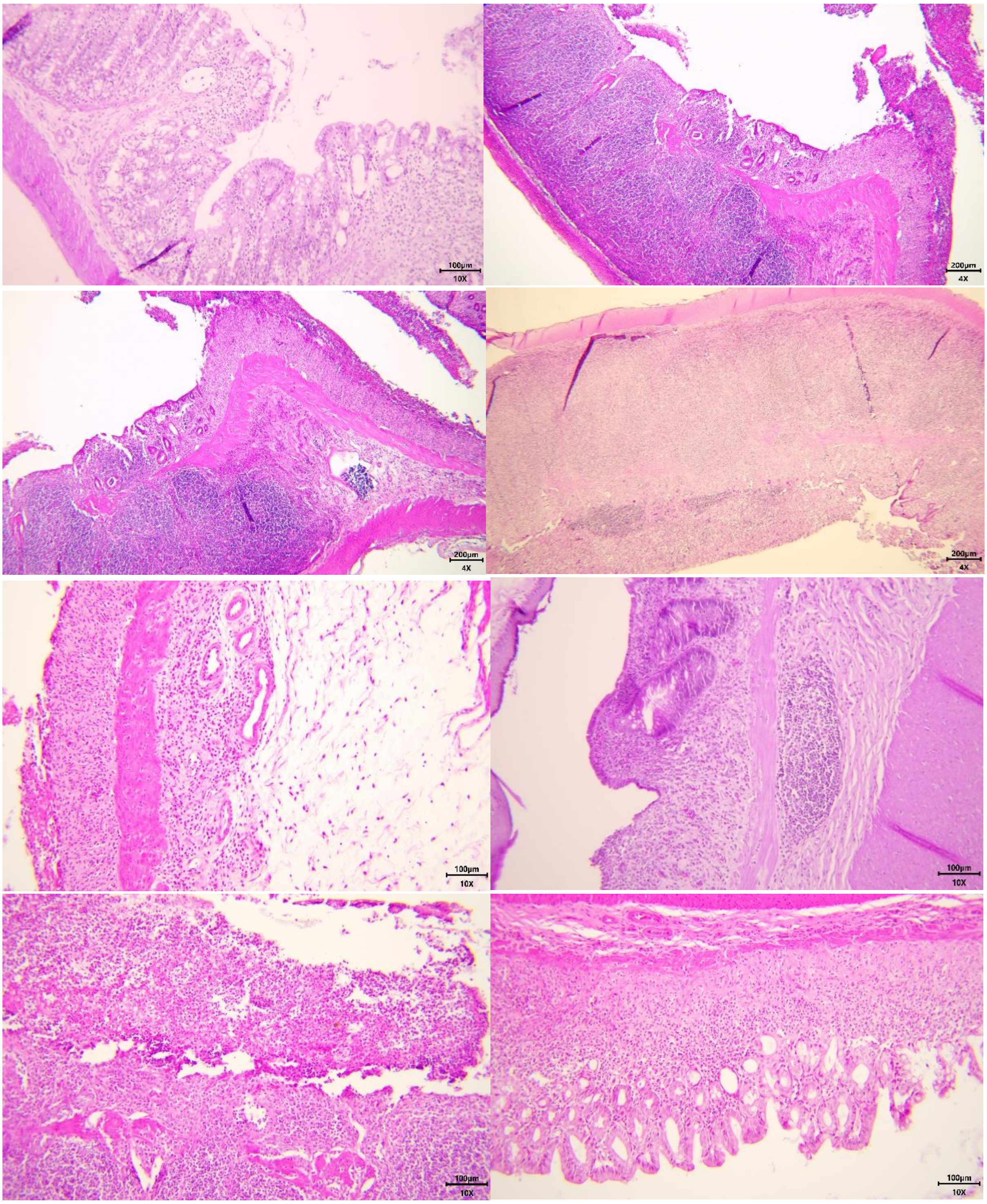

Supplementary Figure S3 - Histological examination of intestinal tissue from DSS group. Formalin-fixed preparation of the distal intestine of an experimental animal. Each figure corresponds to a histological examination of an intestine fragment of animals of the DSS group. Hematoxylin-eosin staining. The samples were evaluated by light microscopy at magnifications of X4 and X10 (Zeiss Primo Star, China)
