# Supplementary material for: Bacteroides vesicles promote functional alterations in the gut microbiota composition
Source: Microbiol Spectr. 2024 Sep 30;12(11):e00636-24. doi: 10.1128/spectrum.00636-24 (PMC11537023; doi:10.1128/spectrum.00636-24)
Supplement: Supplemental material — Fig. S5 and S6; legends. [file spectrum.00636-24-s0005.docx]

**Supplementary information**

**Table S1: Histology and disease activity indexes.** Histology and DAI indexes, obtained during intestine histology examination and animals’ observation

**Table S2: Metabolomic data.** Metabolomic data obtained from control, DSS and DSS+OMV groups

**Table S3: Sequencing data.** Sequencing data, obtained from control, DSS and DSS+OMV groups





**Supplementary Figure S1.** TEM image of the isolated *Bacteroides fragilis* OMVs. Image size 1260x1260 nm. Images were obtained using a JEM-1400 (Jeol, Tokyo, Japan) transmission electron microscope equipped with a Rio-9 camera (Gatan Inc., Pleasanton, CA, USA) at 120 kV. The sizes of the OMVs were measured using ScanEV software.

**
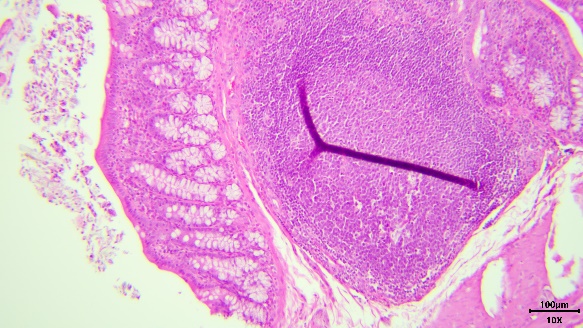

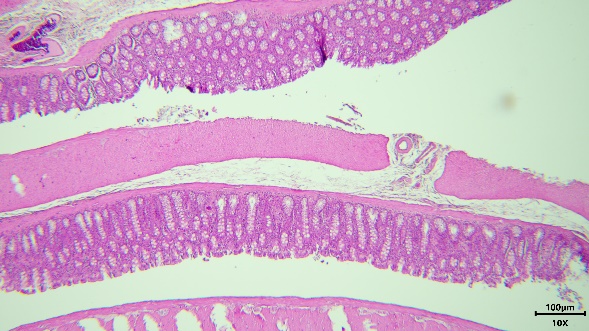
**

**
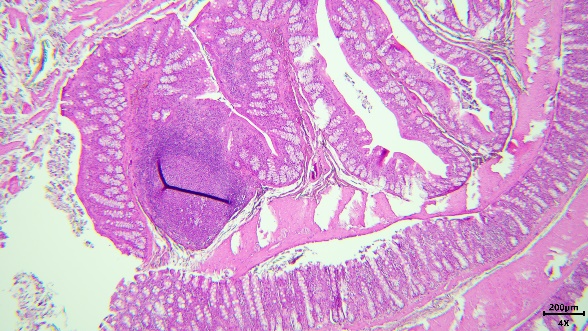

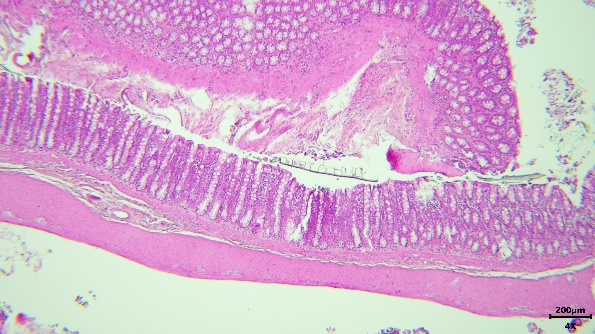
**

**Supplementary Figure S2** - Histological examination of intestinal tissue from control group. Formalin-fixed preparation of the distal intestine of an experimental animal. Hematoxylin-eosin staining. The samples were evaluated by light microscopy at magnifications of X4 and X10 (Zeiss Primo Star, China)

**
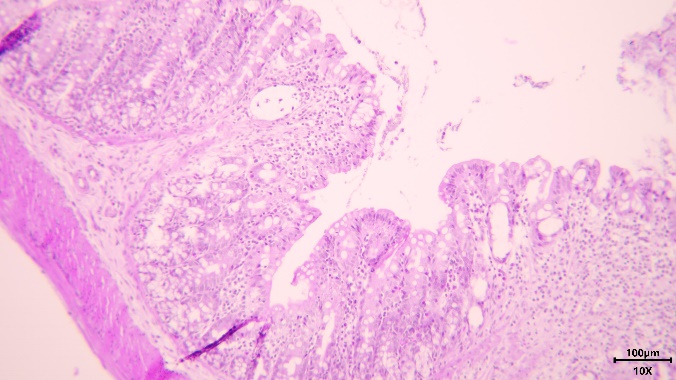

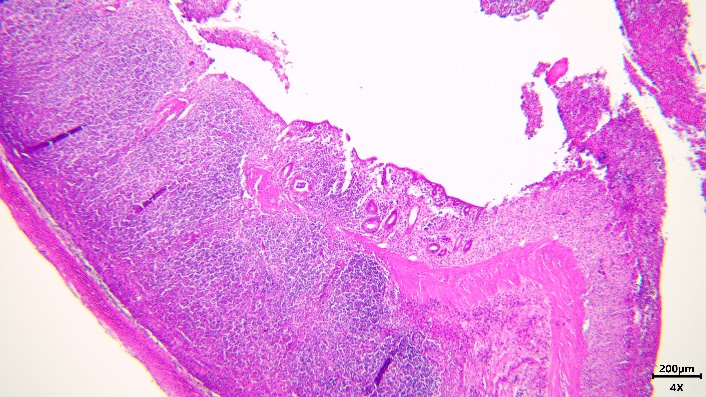

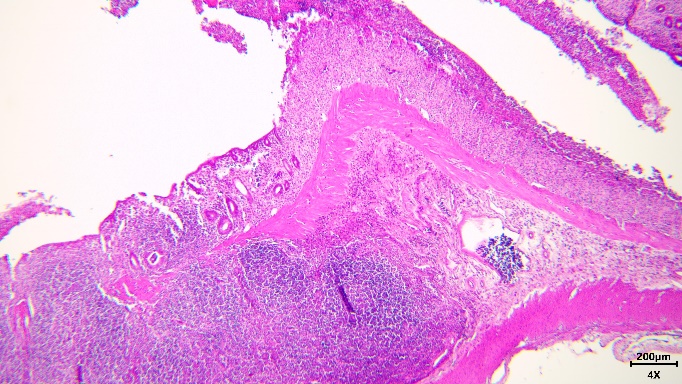

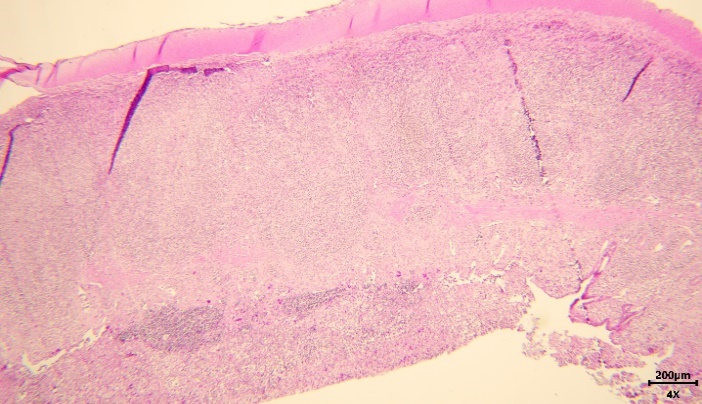

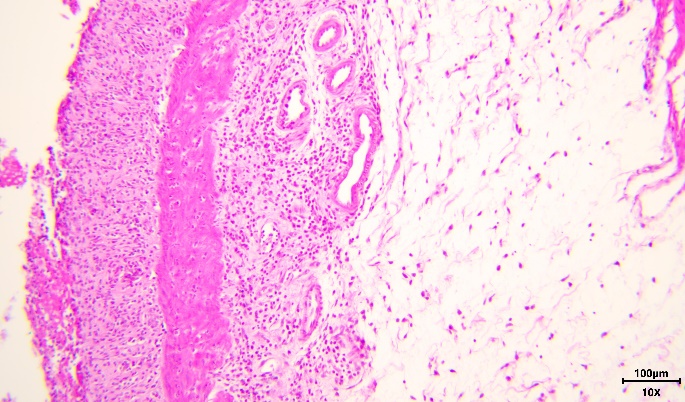

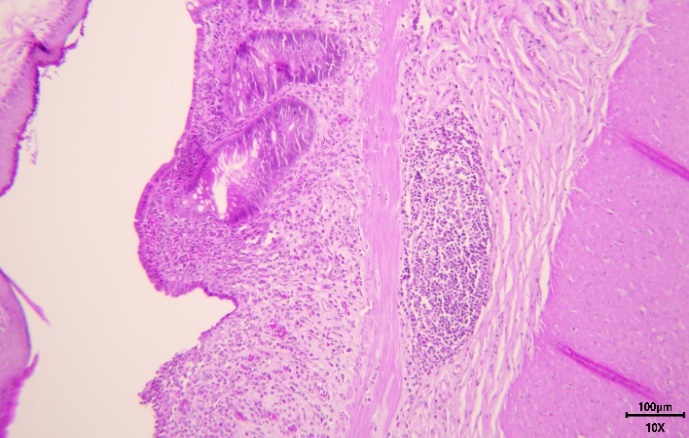

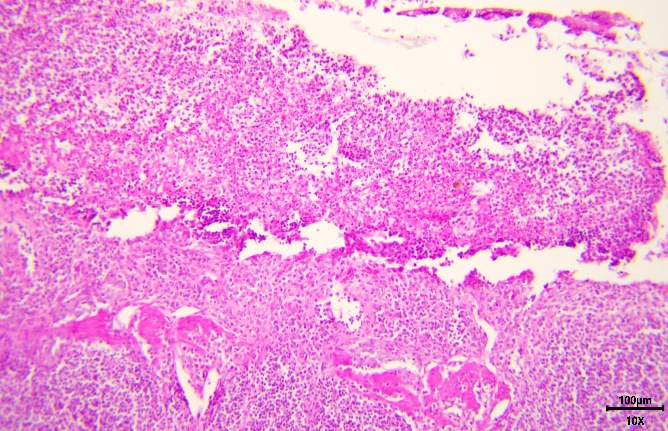

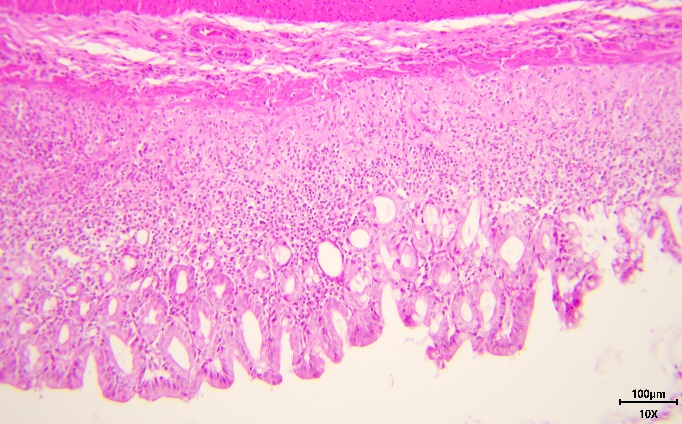
**

**Supplementary Figure S3** - Histological examination of intestinal tissue from DSS group. Formalin-fixed preparation of the distal intestine of an experimental animal. Hematoxylin-eosin staining. The samples were evaluated by light microscopy at magnifications of X4 and X10 (Zeiss Primo Star, China)


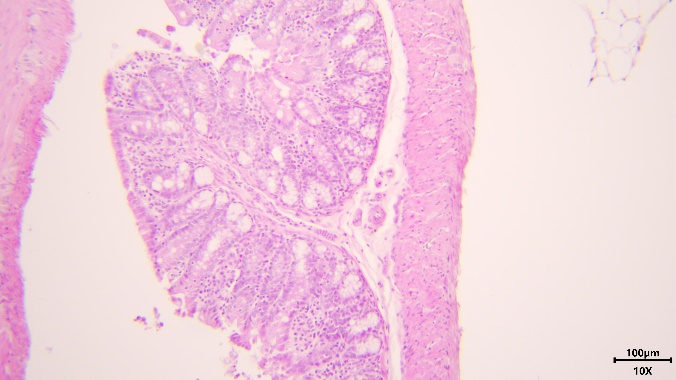

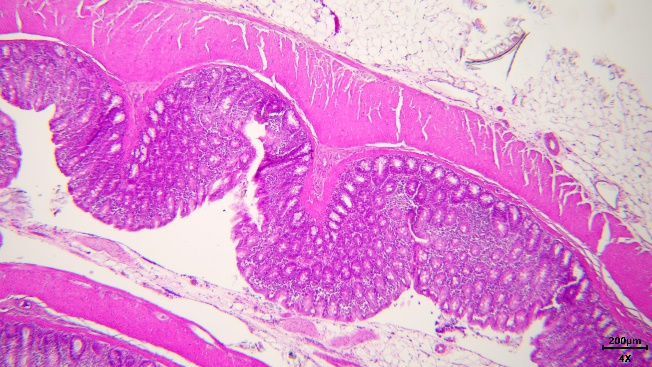

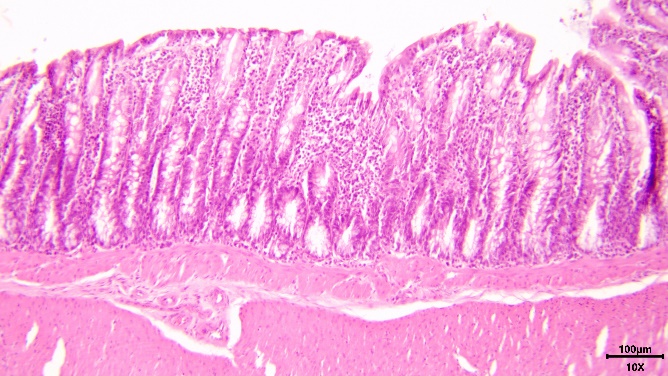

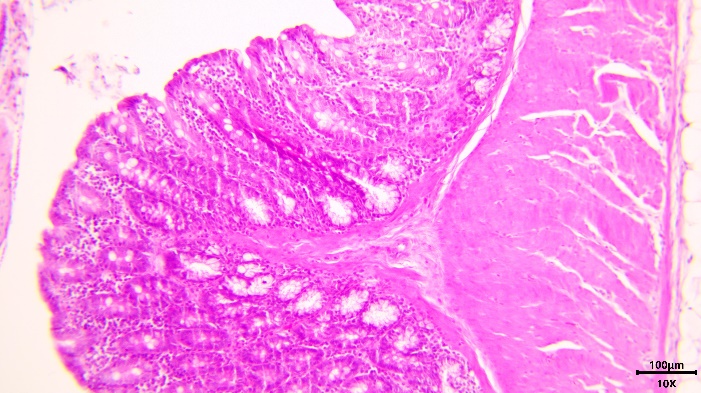

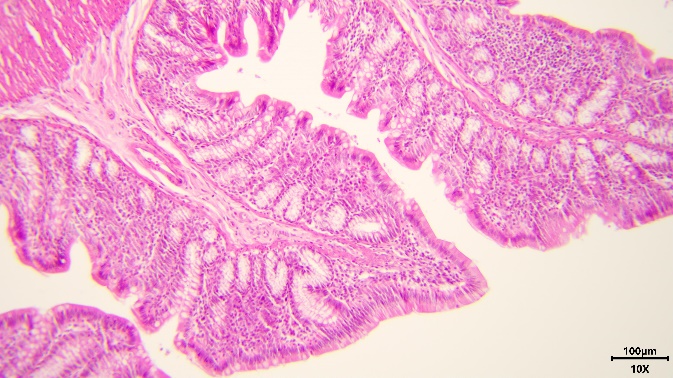

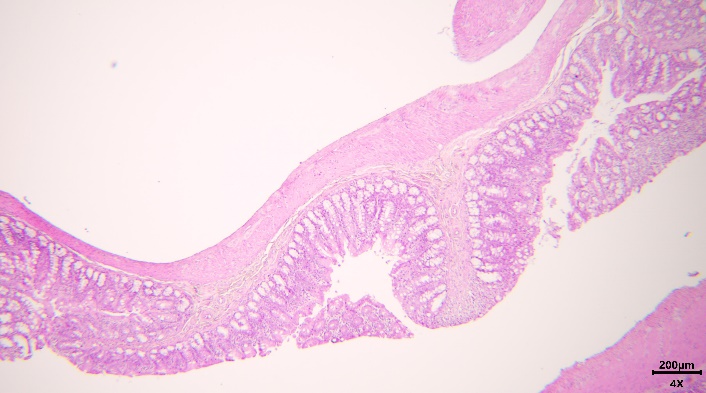

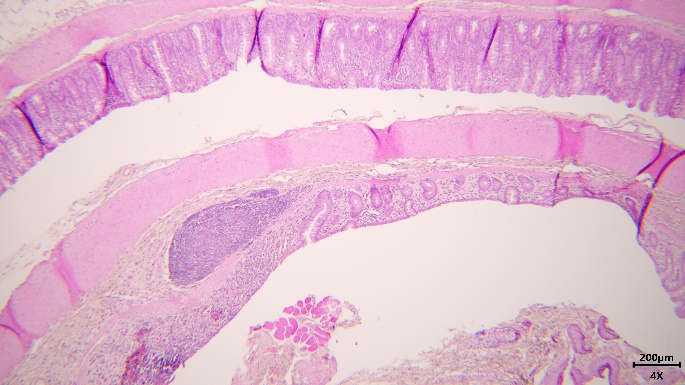

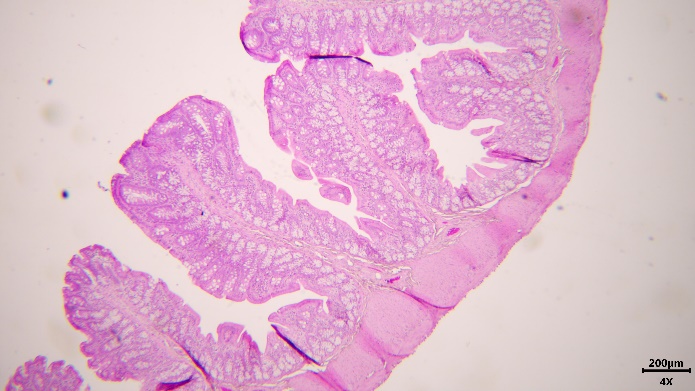

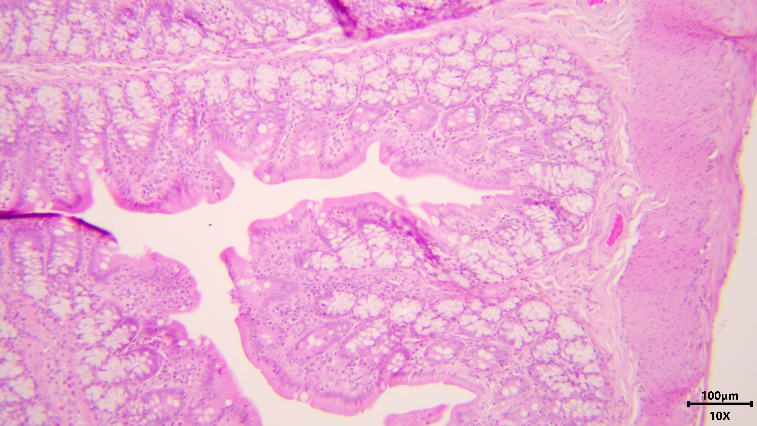


**Supplementary Figure S4** - Histological examination of intestinal tissue from DSS+OMV group. Formalin-fixed preparation of the distal intestine of an experimental animal. Hematoxylin-eosin staining. The samples were evaluated by light microscopy at magnifications of X4 and X10 (Zeiss Primo Star, China)


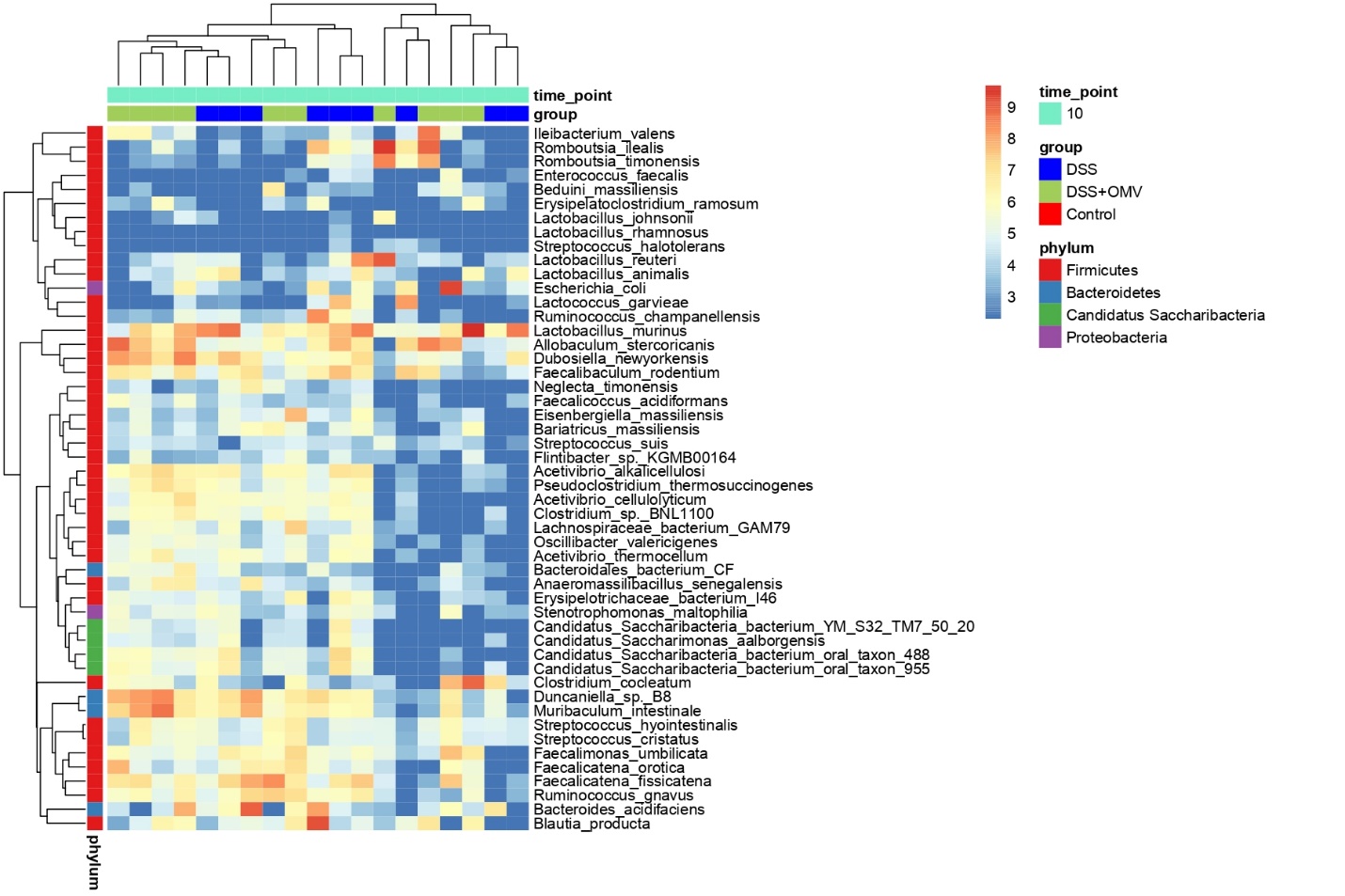

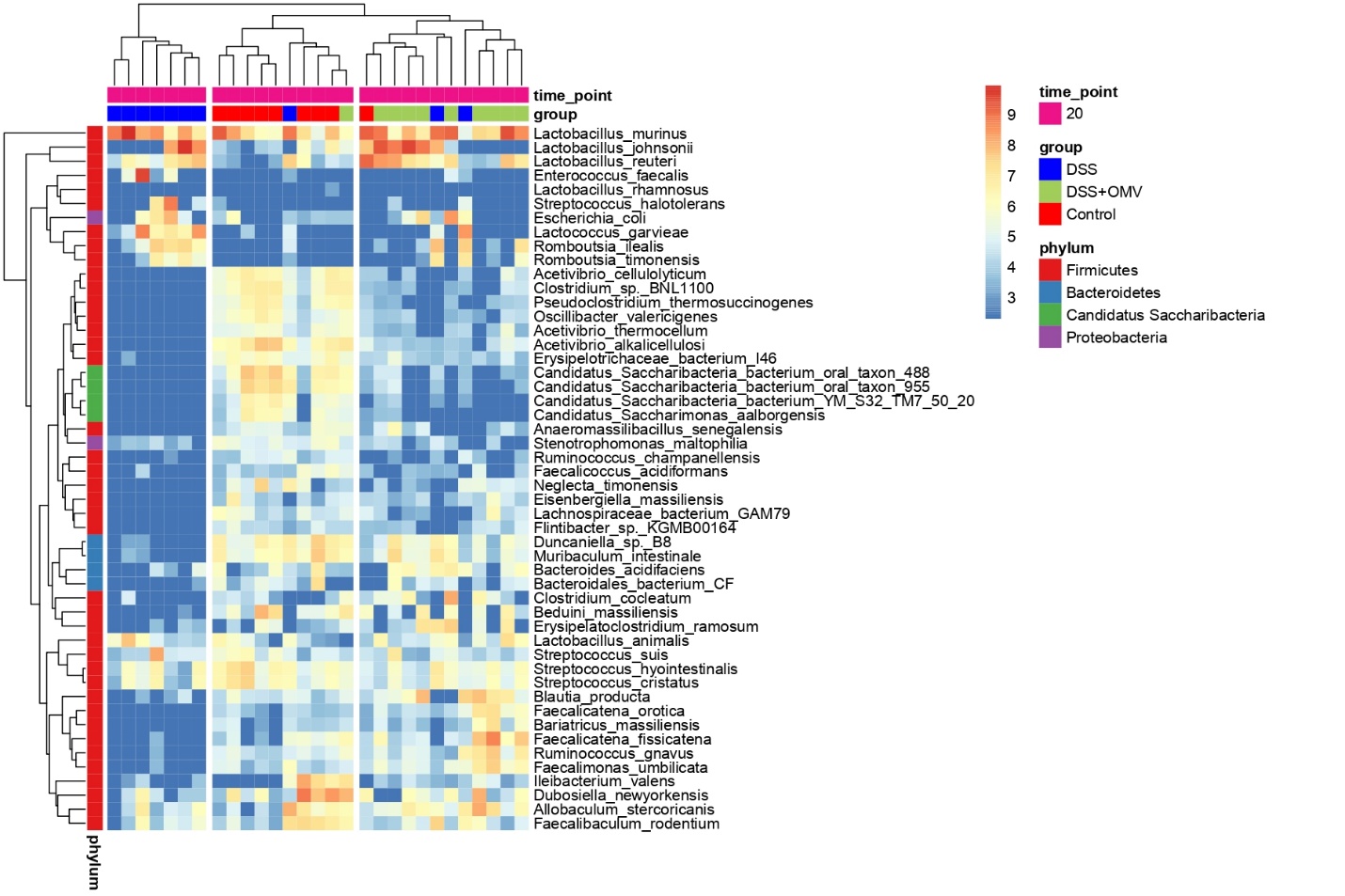


**Supplementary Figure S5**. Bacterial diversity in different time points (10,20 days). The color scale of the heat map shows the abundance of different species. The horizontal axis represents samples, the vertical axis represents different bacterial species.


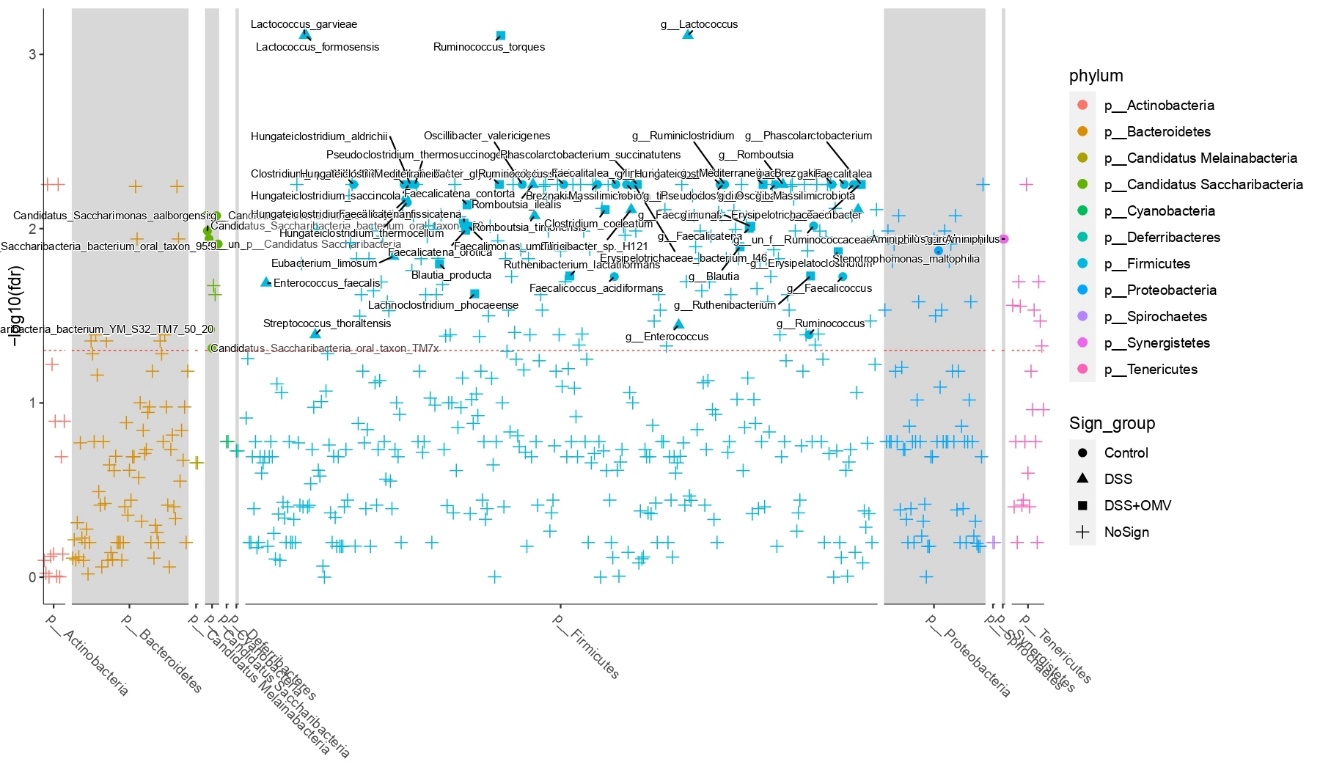


**Supplementary Figure S6**. The Manhattan plot shows significant diversity between experimental groups.
